# Buffalo Hospital of the Sisters of Charity

**Published:** 1853-10

**Authors:** 


*Buffalo Hospital of the Sisters of Charity.*—The accommodations at this hospital have lately been increased by the completion of a new building, making its capacity about one-third greater than heretofore. The original building has also been entirely remodeled. In the convenience and comfort which it now affords for the sick, it will compare favorably with the best institutions of the country, and the opportunities which it offers for clinical instruction are excellent.

The institution remains, as its title imports, under the charge of the Sisters of Charity. In the ministrations of these religious females the homeless and friendless find the sympathy and kindness of a mother or sister added to the means of relief.

We regret to announce the decease of one of the band of sisters attached to the hospital. Sister Annesia, who had been a member of the community only four years, was attacked with typhoid fever on the 28th ult., which terminated fatally on the eighteenth day.

